# Students’ emotional well-being and religiosity during the COVID-19 pandemic- an international study in 7 countries

**DOI:** 10.1192/j.eurpsy.2023.874

**Published:** 2023-07-19

**Authors:** K. H. Karakula, A. Forma, R. Sitarz, J. Baj, D. Juchnowicz, J. Bogucki, W. Tuszyńska-Bogucka, M. L. Tee, C. A. Tee, J. T. Ly-Uson, M. S. Islam, M. T. Sikder, A. H. El-Monshed, A. Loutfy, M. F. Hussain Qureshi, M. Abbas, S. Taseen, M. Lakhani, S. Jayakumar, S. Ilango, S. Kumar, Á. A. Ruiz-Chow, A. Iturbide, D. D. González-Mille, H. Karakula-Juchnowicz

**Affiliations:** 1 I Department of Psychiatry, Psychotherapy and Early Intervention; 2 Department of Forensic Medicine; 3 I Department of Psychiatry, Psychotherapy and Early Intervention; 4 Chair and Department of Anatomy; 5 Department of Psychiatric Nursing; 6 Chair and Department of Organic Chemistry, Faculty of Pharmacy, Medical University of Lublin; 7 Department of Human Sciences, Institute of Psychology and Human Sciences, University of Economics and Innovation, Lublin, Poland; 8 College of Medicine, University of the Philippines Manila, Manila, Philippines; 9 Department of Public Health and Informatics, Jahangirnagar University, Savar, Dhaka, Bangladesh; 10 Department of Psychiatric and Mental Health Nursing, Mansoura University, Mansoura, Egypt; 11 Nursing Department, College of Health and Sport Sciences, University of Bahrain, Manama, Bahrain; 12 Department of Pediatric Nursing, Faculty of Nursing, Beni-Suef University, Beni-Suef, Egypt; 13 Ziauddin Medical University, Zaiuddin; 14 Karachi Medical and Dental College, Karachi, Pakistan; 15 Department of Basic Medical Sciences, Al Majmaah University, Majmaah, Saudi Arabia; 16 Madha Medical College and Research Institute, Chennai, India; 17 Centro Médico ABC, Mexico City, Mexico

## Abstract

**Introduction:**

There are no conclusive findings about the possible protective role of religion on students’ mental health during the COVID-19 pandemic. Therefore, more research is needed.

**Objectives:**

The purpose of this study was to assess the relationship between the level of emotional distress and religiosity among students from 7 different countries during the COVID-19 pandemic.

**Methods:**

Data were collected by an online cross-sectional survey that was distributed amongst Polish (N = 1196), Bengali (N = 1537), Indian (N = 483), Mexican (N = 231), Egyptian (N = 565), Philippine (N = 2062), and Pakistani (N = 506) students (N **=** 6642) from 12th April to 1st June 2021. The respondents were asked several questions regarding their religiosity which was measured by The Duke University Religion Index (DUREL), the emotional distress was measured by the Depression, Anxiety, and Stress Scale-21 (DASS-21).

**Results:**

Egypt with Islam as the dominant religion showed the greatest temple attendance (organizational religious activity: M=5.27±1.36) and spirituality (intrinsic religiosity: M=5.27±1.36), p<0.0001. On another hand, Egyptian students had the lowest emotional distress measured in all categories DASS-21 (depression: M=4.87±10.17, anxiety: M=4.78±10.13, stress: M=20.76±11.46). Two countries with the dominant Christian religion achieved the highest score for private religious activities (non-organizational religious activity; Mexico: M=3.94±0.94, Poland: M=3.63±1.20; p<0.0001) and experienced a moderate level of depressive symptoms, anxiety, and stress. Students from Mexico presented the lowest attendance to church (M=2.46±1,39) and spirituality (M=6.68± 3.41) and had the second highest level of depressive symptoms (M=19.13±13.03) and stress (M=20.27±1.98). Philippines students had the highest DASS-21 score (depression: M=22.77±12.58, anxiety: M=16.07±10.77, stress: M=4.87±10.08) and their level of religiosity reached average values in the whole group. The performed regression analysis confirmed the importance of the 3 dimensions (organizational religious activity, non-organizational religious activity, intrinsic religiosity) of religiosity for the well-being of students, except for the relationship between anxiety and private religious activities. The result was as presented for depression: R^2^=0.0398, F(3.664)=91.764, p<0.0001, SE of E: 12.88; anxiety: R^2^=0.0124, F(3.664)=27.683, p<0.0001, SE of E: 10,62; stress: R^2^= 0.0350, F(3.664)=80.363, p<0.0001, SE of E: 12.30.

**Conclusions:**

The higher commitment to organizational religious activity, non-organizational religious activity, and intrinsic religiositywas correlated with the lower level of depressive symptoms, stress, and anxiety among students during the COVID-19 pandemic, but taking into account factors related to religiosity explains the level of emotional well-being to a small extent.

**Disclosure of Interest:**

None Declared

